# Large Adult Spinal Diffuse Midline Histone H3 Lysine27-to-Methionine-Mutant Glioma With Intramedullary and Extramedullary Components Presenting With Progressive Hydrocephalus: A Case Report Highlighting Unique Imaging Findings and Treatment

**DOI:** 10.7759/cureus.15333

**Published:** 2021-05-30

**Authors:** Evan H Einstein, David Bonda, Hossein Hosseini, Asaff Harel, Joshua D Palmer, Pierre Giglio, Rahul Barve, Megan Gould, Russell R. Lonser, Randy S D'Amico

**Affiliations:** 1 Neurosurgery, Lenox Hill Hospital, Donald and Barbara Zucker School of Medicine at Hofstra/Northwell, New York, USA; 2 Pathology, Lenox Hill Hospital, Donald and Barbara Zucker School of Medicine at Hofstra/Northwell, New York, USA; 3 Neurology, Lenox Hill Hospital, Donald and Barbara Zucker School of Medicine at Hofstra/Northwell, New York, USA; 4 Radiation Oncology, The Ohio State University Comprehensive Cancer Center, Arthur G. James Cancer Hospital and Richard J. Solove Research Institute, Columbus, USA, Columbus, USA; 5 Neuro-oncology, The Ohio State University Wexner Medical Center, Columbus, USA; 6 Neurosurgery, The Ohio State University Wexner Medical Center, Columbus, USA

**Keywords:** glioma, spinal cord tumor, diffuse midline glioma, high-grade glioma, h3 k27m

## Abstract

Diffuse midline glioma with histone H3 lysine27-to-methionine mutation (H3 K27M mutation) is a rare, aggressive tumor that is designated as World Health Organization (WHO) grade IV regardless of histologic features. Preoperative diagnosis remains challenging due to limited evidence regarding distinctive clinical and imaging characteristics. We describe the case of a young woman who presented with progressively worsening headaches due to communicating hydrocephalus. MR imaging with contrast of the cervical and thoracic spine revealed diffuse leptomeningeal enhancement with focal areas of intramedullary and subarachnoid T2 hyperintensity and enhancement, suggestive of a potential infectious process. Intraoperatively, no epidural pathology was identified, and with the differential diagnosis remaining broad, a second procedure was conducted involving intradural exploration and biopsy of a lesion. This was then identified as a diffuse midline glioma with H3 K27M mutation. The nonfocal clinical presentation in the setting of communicating hydrocephalus as well as the significant exophytic tumor growth and imaging findings made the initial diagnosis unique and challenging. This case, therefore, emphasizes the rare presentation of this tumor, and the need for further understanding of the clinical and imaging characteristics of this disease as well as the need for effective therapeutics.

## Introduction

Primary spinal cord tumors are rare neoplasms that lead to significant morbidity and mortality without treatment [1]. The incidence of both malignant and nonmalignant primary spinal cord tumors has been estimated at 0.97 per 100,000 people [2]. Intramedullary spinal cord tumors, including ependymomas, astrocytomas, and hemangioblastomas, are the rarest of these neoplasms [3]. These lesions can be particularly difficult to detect and can also be difficult to distinguish on imaging [4].

Diffuse midline glioma with histone H3 lysine27-to-methionine mutation (H3 K27M mutation) is a novel entity, recently added to the 2016 World Health Organization (WHO) classification of central nervous system (CNS) tumors [5], and the radiographic characteristics of this particular tumor are yet to be fully defined [6,7]. In addition, these are aggressive tumors associated with a poor prognosis and designated as WHO grade IV tumors regardless of their histologic features. Evidence indicates that these tumors arise in the thalamus, pons, and spinal cord of children and young adults and are associated with a poor prognosis [8]. New guidelines for the diagnosis and treatment of adult diffuse glioma have recently been published [9], but our understanding of these challenging tumors is still developing. In this case, we describe the rare presentation and imaging features of a young woman who presented with worsening headaches due to progressive hydrocephalus ultimately due to an intradural, intramedullary diffuse midline glioma H3 K27M-mutant of the thoracic spinal cord with an extensive exophytic subarachnoid component.

## Case presentation

**Figure 1:**
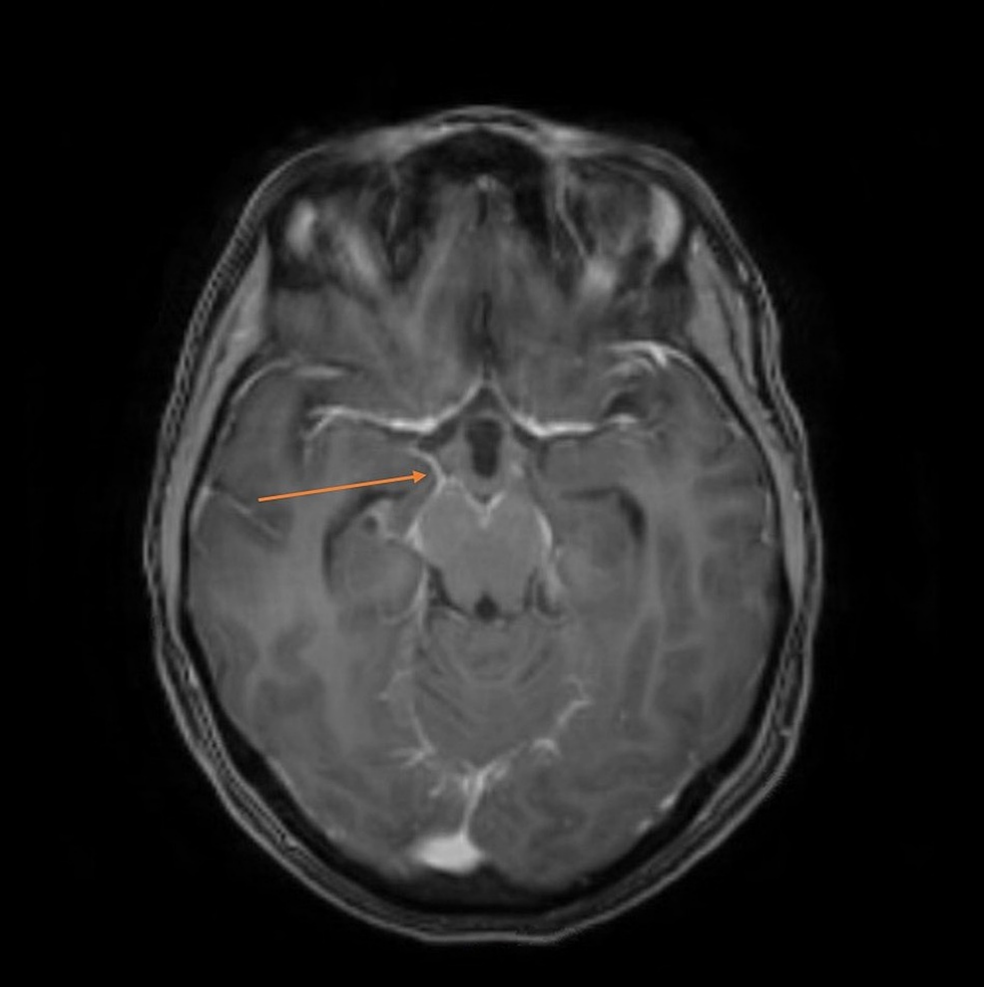
Contrast MRI of the brain demonstrating abnormal enhancement at the ventral brainstem lining the interpeduncular cistern, with temporal horns of the lateral ventricles visible, suggesting hydrocephalus MRI, Magnetic resonance imaging.

A 33-year-old otherwise healthy woman presented to our emergency department after three weeks of headaches, staring spells, associated with whole-body shaking, and several days of visual disturbance. The admission neurological exam was unremarkable. Initial neurological work-up with video-electroencephalogram (vEEG), CT angiogram of the head and neck, noncontrast MRI of the cervical spine, and MR venogram of the head was unrevealing. Noncontrast MRI of the brain revealed mild enlargement of the ventricular system without aqueductal stenosis, suggestive of mild communicating hydrocephalus. Within the first several days of admission, she developed progressively more prominent bilateral lateral rectus palsies, suggestive of increased intracranial pressure. Lumbar puncture for cerebrospinal fluid (CSF) analysis revealed an opening pressure of 30 mmHg, 11 nucleated cells/μL, markedly elevated protein above the limit of quantification (>600 mg/dL), elevated angiotensin-converting enzyme (5.6 U/L), and negative cultures. Contrast MRI of the brain demonstrated abnormal enhancement at the ventral brainstem lining the interpeduncular cistern, at the internal auditory canals along the path of cranial nerves seven and eight, along the trigeminal nerves, and around the cervical spinal cord (Figure 1). Neurosarcoidosis was considered, but a CT of the chest and a gallium scan were unrevealing. Ultimately, based on the leptomeningeal enhancement pattern on MRI and the CSF findings, viral meningitis was considered most likely. Intravenous acetazolamide and dexamethasone were initially trialed with mild improvement of symptoms.

**Figure 2:**
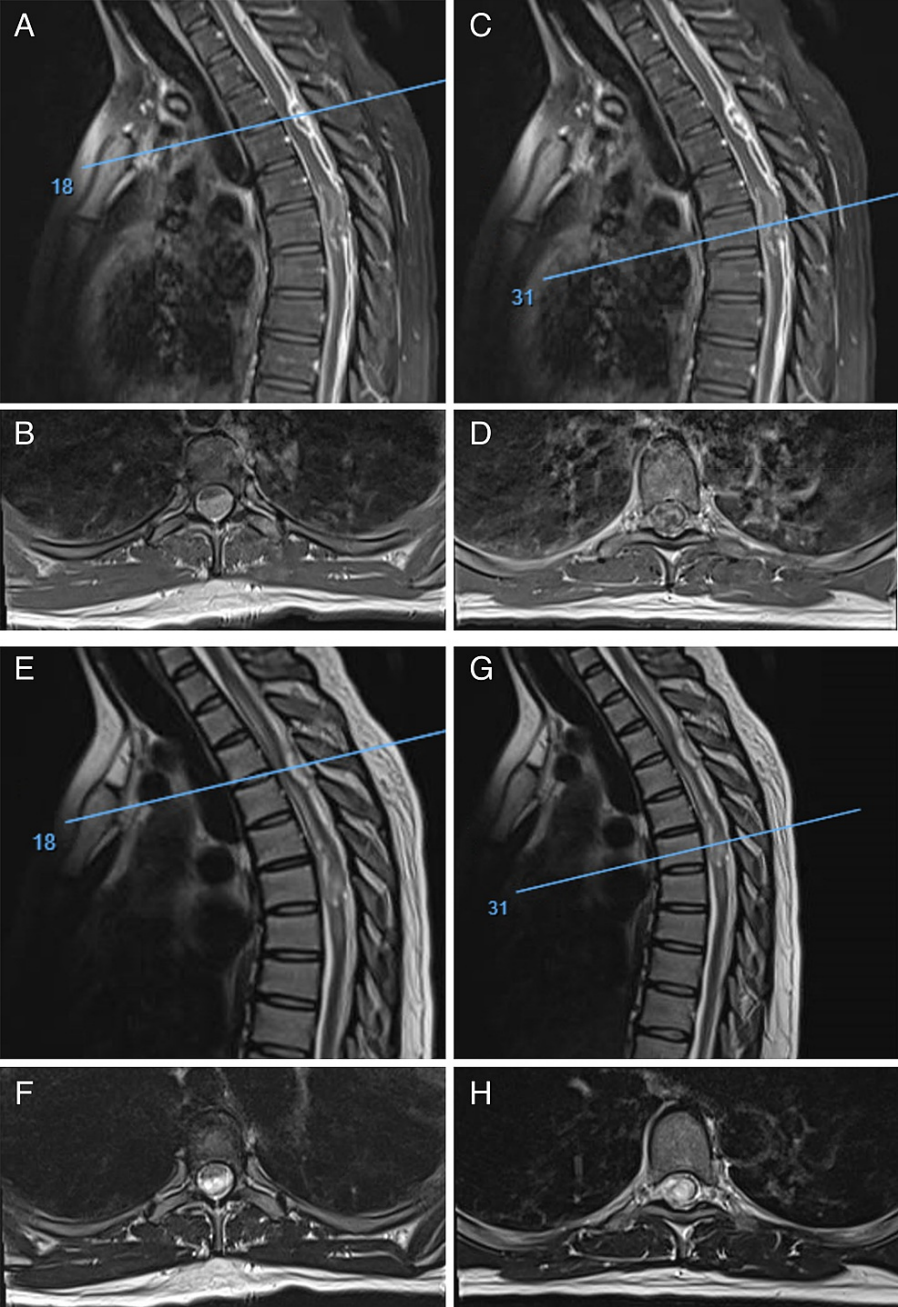
(A) and (B) T1-weighted sagittal MRI with IV contrast of the thoracic spine demonstrating the diffuse, heterogeneous leptomeningeal enhancement with loculated dorsal mass impinging the thecal sac (A) and intramedullary, exophytic mass (C). (B) Axial image of the slice indicated in (A) by the blue line. (D) Axial image of the slice indicated in (C) by the blue line. (E) and (G) T2-weighted sagittal MRI without contrast. (F) Axial image of the slice indicated in (E) by the blue line. (H) Axial image of the slice indicated in (G) by the blue line MRI, Magnetic resonance imaging; IV, intravenous.

Later in the admission, she was noted to exhibit right patellar and ankle hyperreflexia. MRI of the cervical and thoracic spine was obtained with and without intravenous (IV) contrast (given the inconclusive CSF and prior brain MRI findings). This revealed diffuse leptomeningeal enhancement surrounding the cervical spinal cord with a large dorsal epidural enhancing lesion beginning at the C7-T1 level and extending to the thoracolumbar junction as well as focal prominence at the T3-T4 level posteriorly and eccentric to the left, causing displacement and compression of the spinal cord anteriorly and to the right (Figures 2A, 2B, 2E, 2F). The lesion at this point measured 8 mm in the anteroposterior (AP) dimension and 2.2 cm in the craniocaudal dimension. There was also another prominent epidural area at the T9 level with cord impingement. At the level of T6, there was an intradural intramedullary lesion (Figures 2C, 2D, 2G, 2H). This expansile lesion demonstrated heterogeneous T2 hyperintensity and peripheral ring-like contrast enhancement on axial imaging and measured 12 mm in AP dimension and 3.2 cm in craniocaudal dimension. A large area of abnormal T2 signal was also seen centrally within the cord measuring 5 mm x 5 mm in axial dimension and extending from T1 to T8 level consistent with edema. Neurosurgery was consulted at that time due to suspected intramedullary and epidural neoplastic or infectious processes. Notably, while the white blood cell count was mildly elevated, the patient remained afebrile with negative erythrocyte sedimentation rate/C-reactive protein (ESR/CRP) and blood cultures. Vital signs were normal. 

The patient was taken to the operating room (OR) for the urgent evacuation of the possible epidural abscess. Intraoperatively, a small T3 to T4 laminotomy was performed with bilateral medial facetectomy. No epidural pathology was identified at that time, and the thecal sac was noted to be full. Intraoperative ultrasound identified intradural pathology. It was unclear at the time whether the lesion was continuous with the intramedullary component arising at T6 or whether it represented exophytic disease or substantially thickened leptomeningeal involvement. Given the lack of neurophysiological monitoring, the patient’s baseline good neurologic function, and broad differential diagnosis, the decision was made to stage the intradural component of the procedure. Two days later, the patient underwent a second procedure involving intradural exploration, biopsy, and debulking of an intradural extramedullary lesion. A frozen section showed distorted cells, and the intraoperative diagnosis was deferred.

Hydrocephalus persisted postoperatively, and the patient became progressively more symptomatic despite treatment with steroids and acetazolamide, with worsening mental status. The patient was taken for ventriculoperitoneal shunt insertion due to communicating hydrocephalus in the setting of leptomeningeal disease. 

**Figure 3:**
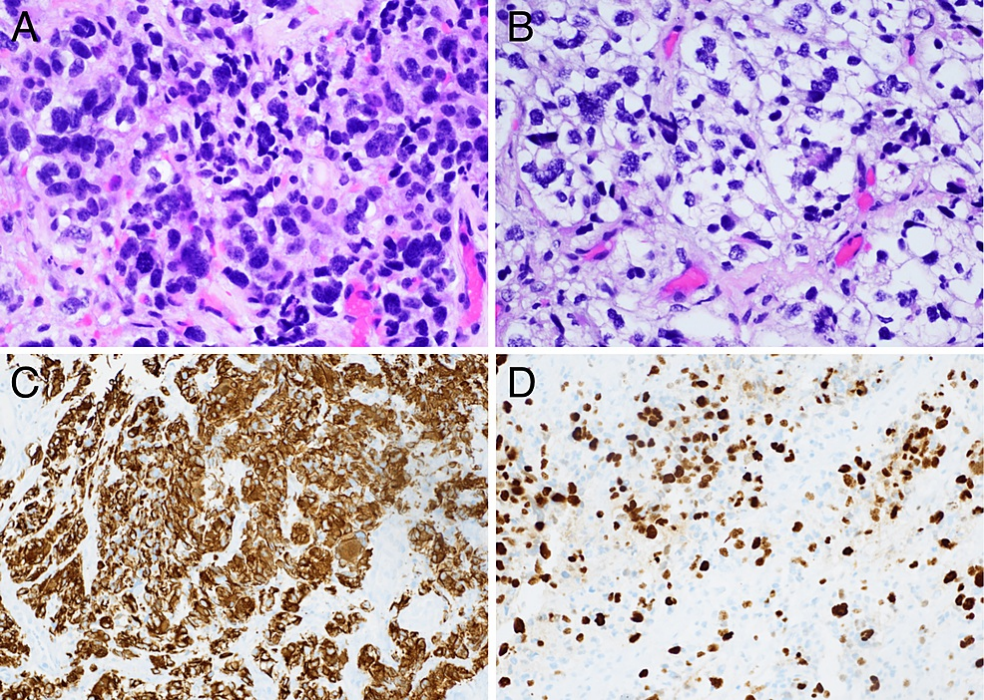
(A and B) H&E (40x) examination showed a cellular neoplasm with focally clear cytoplasm, marked nuclear pleomorphism, and hyperchromasia. Immunohistochemical studies (20x) demonstrating GFAP (C) and Ki-67 (D) H&E, Hematoxylin and eosin; GFAP, glial fibrillary acidic protein.

Final pathological diagnosis was of a diffuse midline glioma with H3 K27M mutation. Histologic examination showed a cellular neoplasm with focally clear cytoplasm, marked nuclear pleomorphism, and hyperchromasia. Immunohistochemical studies demonstrated positive staining of the tumor cells for glial fibrillary acidic protein (GFAP) and *S100 *as well as oligodendrocyte transcription factor (OLIG2), *SOX10*, strong nuclear expression of H3 K27M, and loss of *ATRX *expression. Stain for isocitrate dehydrogenase 1 (IDH1) was negative in the tumor. The *MIB1* proliferation index was 25%-30% (Figure 3). Next-generation sequencing (NGS) performed on paraffin-embedded tissue detected a genomic alteration in *TP53*. O^6^-Methylguanine DNA methyltransferase (MGMT) promoter methylation assay was positive for methylation. Fluorescent in situ hybridization (FISH) was negative for *1p19q *codeletion. 

**Figure 4:**
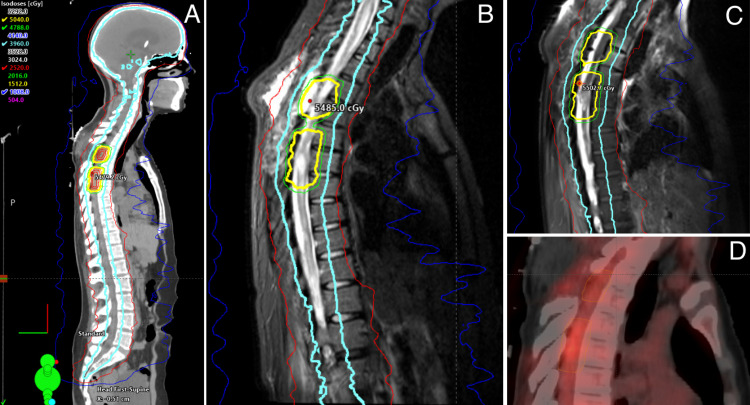
(A) Treatment planning CT (sagittal view) displaying total craniospinal dose of 39.6 Gy with sequential boost dose to a total of 50.4 Gy. (B) Sagittal STIR MRI displaying 50.4 Gy dose. (C) T1 sagittal post-contrast MRI displaying 50.4 Gy dose. (D) PET/CT (sagittal view) displaying increased PET avidity in the spinal cord tumor CT, Computed tomography; STIR, short-TI inversion recovery; MRI, magnetic resonance imaging; PET, positron emission tomography.

The patient was ultimately transferred to her home institution in Ohio to be closer to family. On admission to that facility, the patient was noted to be progressively somnolent. She required intubation, and interrogation of the shunt identified a malfunction likely caused by excessive CSF protein. The shunt system was removed and replaced with an external ventricular drain, which was maintained until the patient could undergo craniospinal radiation. The patient required tracheostomy placement for ventilator wean and also had percutaneous endoscopic gastrostomy (PEG) placement during her hospitalization. She underwent craniospinal radiation to a total dose of 3960 centigray (cGy) with boost; boost 1 was 180 cGy, boost 2 was 900 cGy, involving 5040 cGy to the spinal tumor (Figure 4). This was then followed by the placement of a left ventricular peritoneal shunt. She was significantly impacted with elevated CSF protein during the first week of therapy; she was acutely ill enough that treatment goals were discussed with the family. During radiation, she became more alert and oriented and eventually was able to be extubated after the first two weeks of therapy. She became alert and oriented able to speak with the treatment team, and the CSF protein at the completion of therapy normalized. She was then able to have a ventriculoperitoneal (VP) shunt placed after the completion of therapy.

## Discussion

Diffuse midline glioma with H3 K27M mutation is a distinct subtype of infiltrative glioma [5] with radiographic features that are not yet precisely characterized [6,7]. New guidelines on the diagnosis and treatment of adult diffuse gliomas have recently been published [9], but our understanding of these rare, challenging tumors is still developing. Diagnostic criteria for H3 K27-mutant diffuse midline gliomas currently include a diffuse growth pattern, a midline location (e.g., thalamus, brainstem, spinal cord), and the histone H3 K27M mutation. The mutations in H3 K27M mutants involve lysine 27 in the N-terminal tail of histone H3, which is responsible for broad epigenetic regulation. The mutations are often associated with *TP53 *overexpression and typically exclude mutations suggesting more favorable prognoses including *IDH1 *mutations [10]. The case described in this report involves the presentation and imaging findings of a diffuse midline glioma H3 K27M-mutant that was initially diagnostically challenging, given the patient’s clinical presentation and lack of specific radiographic correlations. 

There is limited literature on the various imaging characteristics of H3 K27M-mutant gliomas, particularly in adults and within the spinal cord itself. Generally, intramedullary spinal tumors often involve expansion of the spinal cord, effacement of the CSF spaces, and varying degrees of contrast enhancement on MR imaging [11]. However, descriptions of MR imaging involving H3 K27M-mutant gliomas are highly variable. In pediatric patients, H3 K27M-mutant gliomas in the cervical spine (C-spine) have demonstrated expansile fluid-attenuated inversion recovery (FLAIR) hyperintense signal within the spinal cord [12]. One study that examined these tumors in adults found that the majority of enhancement patterns were described as ring-like, patchy, and homogenous [6]. On T1-weighted MR images, most lesions demonstrated thin walls with a relatively uniform thickness along capsules that showed homogenous, CSF-like signals. This description contrasts with true glioblastoma lesions, which often present with uneven wall thickness and mixed capsule signals on T1 images. Although the majority of this study examined thalamic primary lesions, the spinal tumor cases showed both no enhancement and irregular peripheral enhancement in long-segment lesions. In the case described above, diffuse leptomeningeal enhancement was described extending along both the C-spine and thoracic spine (T-spine) as well as areas of focal extramedullary, subdural, and subarachnoid prominence, which appeared indistinguishable from the extradural disease on MR imaging. In addition, the focal lesion at the T6 level within the intramedullary intradural thoracic spinal cord was described as having peripheral ring-like enhancement. It is unclear even from intraoperative images whether the intradural extramedullary tumor components arose from substantially thickened leptomeningeal involvement or from exophytic growth. 

Another study specifically examined imaging of diffuse midline gliomas found in the spinal cord of both adults and children [7]. Both H3 K27M-mutant and wild-type tumors were compared using a machine learning-based classification model to ascertain specific identifiable characteristics. Overall, the study concluded that MR imaging features of spinal cord diffuse midline gliomas were heterogenous. However, size was determined to be the most important predictor of the presence of the H3 K27M mutation as compared to wildtype. Analysis also showed that the presence of hemorrhage was the only imaging feature able to predict H3 K27M mutation status; hemosiderin deposition represented as a T2 low signal intensity inside the tumor was only seen in mutant tumors as compared to wildtype.

In previous case reports [13-16], patients with intramedullary primary spinal cord glioblastoma with significant exophytic growth underwent laminectomy followed by radiation therapy with temozolomide. In one case report [14], the patient had intracranial dissemination on follow-up MRI but remained alive for nine months following surgery. In another case report [15], the patient received craniospinal irradiation (CSI) and had an overall survival of three months. To reiterate, management of spinal glioma remains a challenge due to limited research and guidelines for therapy, and further research is necessary to develop optimal treatment strategies.

Standard therapy for H3 K27-mutant diffuse midline gliomas includes fractionated external beam radiotherapy [17]. Surgical resection of diffuse midline glioma typically poses a challenge due to location, and there is limited literature on the surgical treatment of these specific gliomas within the spinal cord. Medical management is also currently limited, and although temozolomide is indicated in adult glioblastoma, its use appears limited in H3 K27M-mutant gliomas [18]. However, the recent use of selective dopamine receptor D2 (DRD2) antagonist ONC201 has demonstrated maintenance of progression-free disease in a small cohort of patients [17], although more data will be required for further understanding of clinical benefit.

## Conclusions

We present a rare case of a diffuse midline H3 K27-mutant spinal cord glioma with highly unusual clinical and radiographic findings. Communicating hydrocephalus, resulting from the leptomeningeal spread and elevated cerebrospinal fluid protein content, produced a poorly localizing clinical picture. The significant exophytic growth, from intramedullary to subdural space within the spinal cord, has only been reported once in the literature. The novelty of this presentation, therefore, made diagnosis challenging. Given our limited understanding of the distinctive imaging features of diffuse midline glioma, it is clear that more work is needed to further elucidate the characteristics of this tumor, particularly in adults and within the spinal cord itself.
